# Supplementary material for: Facelock: familiarity-based graphical authentication
Source: PeerJ. 2014 Jun 24;2:e444. doi: 10.7717/peerj.444 (PMC4081289; doi:10.7717/peerj.444)
Supplement: Supplemental Information 1 [file peerj-02-444-s001.pdf]

Consent

I understand that I have the right to withdraw from this experiment at any time. Full debriefing will be give on completion of the experiment. I understand that I can ask questions at any point. Should I have any further question about this research in future I can contact the researcher using the contact details provided. Complete confidentiality is provided in accordance with The Data Protection Act (1998) and my identity will not be disclosed in connection with this research or its outcome.

I agree to the terms above and give my consent to participate in this study

Print name ______________________________

Signature ______________________________

Date ______________________________
